# Tumours

**Published:** 1919

**Authors:** 


**Tumours.** By Sir J. Bland-Sutton, LL.D. Sixth Edition. Pp. ix., 790. London: Cassell & Company Ltd. 1917. Price 21s. net.

We heartily welcome this new edition of Sir J. Bland-Sutton's well-known work on Tumours, and we feel grateful both to the author and his publishers for bringing out this new edition during a time when the production of almost anything is attended by unusual difficulty. In a review of the sixth edition of this book there is no need again to refer to its excellence, or its special characters, they are both too widely known, but it may be well to call attention to some of the additions to this edition. A very interesting account is given of the effects of morbid conditions of the pituitary body, both enlargements and tumours. Tumours which have been described as arising in the carotid body are also described. The enlargement of the thymus gland, which may cause such serious symptoms in infants, and its operative treatment, is also considered. Perhaps it may be thought that as this is not due to the growth of any tumour, it is not quite in place in this work. But Sir J. Bland-Sutton in the old edition of his book includes such morbid conditions as a distended gall-bladder and a hydrocele of the tunica vaginalis, and in this new edition two new diseases not really tumour growths are included—hydrocephalus and spina bifida. We think this unfortunate, as one does not naturally turn to a work on tumours for a description of these conditions. To the numerous and excellent illustrations in the earlier editions of this book eighty new ones are added in this edition. We are sorry to notice the omission in the new edition of the illustration of a skiagram of an odontome, which was a valuable addition to the chapter on odontomas in the last edition.

